# Natural bioactive compounds in Alzheimer's disease: From the perspective of type 3 diabetes mellitus

**DOI:** 10.3389/fnagi.2023.1130253

**Published:** 2023-03-16

**Authors:** Juan Huang, Nanqu Huang, Qianhua Mao, Jingshan Shi, Yu Qiu

**Affiliations:** ^1^Department of Pharmacology and Chemical Biology, Shanghai Jiao Tong University School of Medicine, Shanghai, China; ^2^Key Laboratory of Basic Pharmacology of Ministry of Education and Joint International Research Lab of Ethnomedicine of Ministry of Education, Zunyi Medical University, Zunyi, Guizhou, China; ^3^School of Public Health, Zunyi Medical University, Zunyi, Guizhou, China; ^4^National Drug Clinical Trial Institution, Third Affiliated Hospital of Zunyi Medical University (The First People's Hospital of Zunyi), Zunyi, Guizhou, China

**Keywords:** Alzheimer's disease, natural bioactive compounds, type 2 diabetes mellitus, type 3 diabetes mellitus, antidiabetic medication, traditional Chinese medicines, polyphenols, alkaloids

## Abstract

There is a close relationship between Alzheimer's disease (AD) and diabetes mellitus (DM), and the link between the two is often referred to as type 3 diabetes mellitus (T3DM). Many natural bioactive compounds have shown the potential to treat AD and diabetes. We mainly review the polyphenols represented by resveratrol (RES) and proanthocyanidins (PCs) and alkaloids represented by berberine (BBR) and *Dendrobium nobile* Lindl. alkaloids (DNLA) from the perspective of T3DM to review the neuroprotective effects and molecular mechanisms of natural compounds in AD.

## Introduction

Alzheimer's disease (AD) is a common neurodegenerative disease that brings a heavy burden to patients, families, and society (Jia et al., 2018; Alzheimer's Disease International, 2019). Although aducanumab, lecanemab and GV971 brought new hope for treating AD in recent years, these drugs are still controversial (Biogen, 2019; Wang et al., 2019; The Lancet, 2022). Therefore, actively exploring the pathogenesis and treatment of AD has important scientific significance and social value. Finding natural bioactive compounds from plants to treat diseases has a long history and has achieved a lot of brilliant results. For example, quinine extracted from *Cinchona calisaya* has antimalarial effects, and paclitaxel extracted from the Pacific yew has anti-cancer effects. In terms of AD treatment, there is also Huperzine-A from a plant called Chinese club moss (*Huperzia serrata*) that has been put into clinical use in AD therapy. This reminds us that natural bioactive compounds are a treasure trove of drugs that could potentially be used in the treatment of AD.

With the continuously deepening understanding of AD, the close connection between diabetes mellitus (DM) and AD has attracted more and more attention. The results of epidemiology show that elderly people with diabetes have a higher risk of AD than peers with non-diabetes. DM, especially type 2 diabetes mellitus (T2DM), increases the risk of AD (Tolppanen et al., 2013). Basic studies have also found that 3 × Tg-AD mice showed age-dependent impaired glucose tolerance (Vandal et al., 2015). The brain of mice with DM showed hyperphosphorylation of tau levels and accumulation of β-amyloid (Aβ) plaque (Oliveira et al., 2021). Aβ is a product of amyloid precursor protein (APP) and is degraded or cleared by an insulin-degrading enzyme (IDE) (Lee et al., 2017; Díaz et al., 2022; Imbimbo et al., 2023). Tau primarily provides stabilization to microtubules in the part of axons and dendrites and shows a loss of microtubule binding for the hyperphosphorylation in AD. Moreover, the glycogen synthase kinase 3β (GSK3β) and mitogen-activated protein kinases affect phosphorylate tau (p-tau) and the formation of neurofilaments (Rawat et al., 2022). Kinesin-I heavy chain (KIF5B) and histone deacetylase (HDAC) are involved in tau homeostasis in AD (Simões-Pires et al., 2013; Selvarasu et al., 2022), which relates to the ubiquitin–proteasome system primarily clearing pathological tau and the autophagy–lysosome pathway degrading tau at the late stage of the formation of neurofibullary tangles (NFTs) (Rawat et al., 2022). Some compounds, such as phenolics, flavonoids, and alkaloids, have the potential to treat AD by targeting tau (Durairajan et al., 2022). Apart from pathological features, T2DM and AD share molecular mechanisms and potential targets, including insulin/IGF-1 signaling, GSK3β, inflammation, mitochondrial dysfunction, and the ApoE4 allele (Hamzé et al., 2022). Insulin resistance and/or deficiency have complex interactions with mitochondrial dysfunction, Aβ deposition, tau hyperphosphorylation, etc., thereby promoting the occurrence and development of AD (Zhang et al., 2018). Therefore, the hypothesis was put forward that AD in connection with type 2 diabetes mellitus is considered to be “type 3 diabetes mellitus (T3DM)” (Steen et al., 2005).

Moreover, some clinical studies have shown that anti-diabetic medications have a certain role in the treatment of cognitive dysfunction caused by DM (Akimoto et al., 2020). Many extracts from traditional Chinese herbs can be used for both DM and AD. Therefore, there is a search for anti-AD drugs based on these mechanisms in natural bioactive compounds. It is helpful for patients with AD with elevated blood glucose (BG) as the early manifestation or with diabetes.

There are many bioactive compounds that improve BG or play a role in neurological protection. For example, protopine (PRO) may have utility in the treatment of T2DM (Moser et al., 2014). In another example, the Bromo-PRO (PRO-Br), a novel PRO derivative, promotes the clearance of pathogenic tau by enhancing the expression of heat shock protein 70 and lysosome-associated membrane protein 2A (Sreenivasmurthy et al., 2022a,b). Similarly, tetrandrine (TET) as a P-glycoprotein (P-gp) inhibitor works in T2DM (Shan et al., 2013) and reduces tau aggregation by rescuing lysosomal Ca^2+^ homeostasis in AD (Tong et al., 2022). Here, we mainly review the polyphenols represented by resveratrol (RES) and proanthocyanidins (PCs), alkaloids represented by berberine (BBR), and *Dendrobium nobile* Lindl. alkaloids (DNLA) from the perspective of T3DM to review the neuroprotective effects and molecular mechanisms of natural compounds in AD.

## Polyphenols

Polyphenols are widely found in grapes, tea, cocoa, and other plants, including flavonoids, tannins, phenolic acids, and anthocyanins. The common feature of polyphenolic compounds is their good antioxidant activity. Many natural bioactive compounds with anti-AD potential are polyphenolic compounds. Here, we choose RES and PCs to review the role and mechanism of these natural bioactive compounds in AD from the perspective of T3DM.

### Resveratrol

Resveratrol is a non-flavonoid polyphenolic compound. RES and its derivatives are mainly found in plants such as the genus *Vitis* L, genus *Polygonum*, genus *Arachis*, and genus *Veratrum*. It is an antioxidant produced by many plants when stimulated (Huang J. et al., 2020). Studies have shown that RES has anti-inflammatory, anti-oxidation, anti-aging, and other effects (Moussa et al., 2017; Huang J. et al., 2020). In particular, RES also has the potential to regulate insulin signaling pathways, improve BG, and improve cognitive function.

In DM, RES shows significant therapeutic potential in ameliorating key symptoms of DM as well as the other concurrent indicators. In clinical trials, RES modulates BG (Szkudelska et al., 2021), HA1c, systolic blood pressure, total cholesterol (Bhatt et al., 2012), and low-density lipoprotein levels in patients with T2DM (Asadi et al., 2017). Furthermore, longer RES intervention time (≥ 6 months) increases total antioxidant status levels in a dose-dependent manner in patients with T2DM (Bo et al., 2017). The anti-diabetic effect of RES is mainly manifested as improving the level of insulin resistance, enhancement of glucose uptake and metabolism, and preservation of islet β-cells (Szkudelski and Szkudelska, 2011). RES has some insulin-sensitizing effects, mainly by activating silent information regulator 1 (SIRT1), AMP-activated protein kinase (AMPK), and forkhead box protein O1 (FOXO1) to regulate NADPH, reactive oxygen species (ROS), and peroxisome proliferators-activated receptors (PPAR) levels, thereby improving mitochondrial function and oxidative stress and relieving insulin resistance (Huang D. D. et al., 2020). Moreover, RES may also increase glucose uptake and metabolism by activating insulin receptor substrate (IRS), PI3K/Akt, AMPK signaling pathways, and endogenous GLUT4 translocation (Chi et al., 2007; Sin et al., 2015). In addition, RES can protect islet β-cells by inhibiting the inflammatory and reducing ROS levels, which is related to the regulation of SIRT1, AMPK, FOXO1, Nrf2, and NF-κB (Zheng et al., 2013; Guo et al., 2014). In conclusion, the improvement effect of RES on diabetes is related to the activation of SIRT1 and insulin-related signaling pathways, thereby inhibiting inflammation and oxidative stress, and improving mitochondrial function.

In AD, RES may be effective in the prevention or treatment. In clinical trials (NCT01504854), oral RES can ameliorate cognitive function in subjects with mild to moderate AD, which involves the regulation of neuroinflammation (Moussa et al., 2017). It restores abnormally high levels in the proteolytic activity of the ubiquitin-proteasome system (Labban et al., 2021). On the one hand, it increases levels of neurotrophins, synaptic markers, and SIRT. On the other hand, it decreases the accumulation of Aβ oligomers, the markers of apoptosis, autophagy, endolysosomal degradation, and ubiquitination in the brains of 3 × Tg (Broderick et al., 2020). *In vitro*, 50 μmol/L RES for 12 h significantly reduces the levels of pS396 and pS199 by regulating CDK5 and GSK-3β activity in the cell (Fang et al., 2021). In another Aβ-induced cell model, RES attenuates Aβ-mediated microglial inflammatory responses by inhibiting the TLR4, NACHT, LRR, NLRP3, and STAT cascade signaling pathways (Capiralla et al., 2012; Feng and Zhang, 2019). Furthermore, it also reduces microglia-dependent Aβ toxicity by activating SIRT1 and inhibiting NF-κB and microglial overactivation (Chen et al., 2005; Steiner et al., 2016; Locatelli et al., 2018). Therefore, Res can delay or prevent key pathological indicators of AD, abnormal Aβ, and tau through anti-oxidation, anti-inflammatory function, and improving mitochondrial function, ultimately improving the spatial learning and memory ability in AD. These functions are similar to its basic mechanism of anti-diabetes and are related to activating SIRT1 and insulin-related signaling pathways.

### Proanthocyanidins

Proanthocyanidins, a class of polyphenolic compounds, are widely distributed in plants, such as grapes, black wolfberry, and blueberry (Maria, 2014). PCs have antioxidants and anti-cancer, anti-inflammatory, cardioprotective, and antibacterial effects. They are promising in the treatment of chronic metabolic diseases such as cancer, DM, and cardiovascular disease (Valencia-Hernandez et al., 2021). They also play a protective role in neurodegenerative diseases, such as AD and Parkinson's disease (Zhang et al., 2019; Zhao et al., 2019).

In DM, PCs improve the damage induced by the diet in insulin-resistant models, glycemia, and insulin sensitivity. PCs target several tissues involved in glucose homeostasis. In insulin-sensitive tissues, PCs modulate glucose uptake and lipogenesis and improve their oxidative/inflammatory state. In the pancreas, PCs modulate insulin secretion and production and β-cell mass, although the available results are divergent (Gonzalez-Abuin et al., 2015). Since PCs may be extracted from different plants, they can also be differentiated into different PCs, such as apple procyanidins (APCs) and lotus seedpod oligomeric PC (LSOPC). However, their hypoglycemic effects are not the same. Specifically, APCs ameliorate insulin resistance by improving hepatic insulin signaling through the suppression of hepatic inflammation in ob/ob mice (Ogura et al., 2016). Meanwhile, LSOPC and synbiotics may regulate glucose disposal in peripheral target tissues through the p66Shc-mechanistic/mTOR signaling pathway in high fat and streptozotocin (STZ)-induced diabetes (Li X. et al., 2017). Furthermore, A- and B-type PC oligomers from different cinnamon species also improve insulin sensitivity to decrease BG in T2DM (Lu et al., 2011). In addition, A-type PC oligomers mainly improve insulin concentration in the blood and pancreas, whereas B-type PC oligomers promote lipid accumulation in the adipose tissue and the liver (Chen et al., 2012). In conclusion, although various PCs have different mechanisms of action in DM, what these effects have in common is improving insulin resistance and increasing insulin sensitivity, and anti-inflammatory and anti-oxidative stress are at the core of these effects.

In AD, PCs may promote cognitive function and thus be beneficial to alleviate AD. PCs can enhance synaptic plasticity by upregulating SIRT1 to improve cognition (Michán et al., 2010; Yokozawa et al., 2011). Notably, PCs and some of their metabolites stimulate CREB, acting as a molecular switch from short- to long-term memory, based on the interplay of the CREB-SIRT1 axis (Zhao et al., 2019). Grape seed PCs (GSPCs) improve isoflurane-induced cognitive dysfunction by protecting against perturbing antioxidant enzyme activities and the NR2B/CREB pathway (Gong et al., 2020). In addition, the PCs effectively inhibit the aggregation of human islet amyloid polypeptide (hIAPP) and Aβ through hydrophobic and hydrogen bonding interactions and also dissolve the aged fibrils (Xu et al., 2021). LSOPC inhibits the formation of advanced glycation end-products by scavenging reactive carbonyls, helping to prevent age-associated diseases represented by AD (Wu et al., 2013). Overall, based on the strong physicochemical properties and antioxidant capacity of PCs, PCs have potential use in anti-AD treatments, although they are still a long way from being used in clinical drugs, and are also great as a functional food ingredient.

## Alkaloids

Alkaloids are a type of organic compound containing nitrogen. Most alkaloids are distributed in higher plants, especially in dicotyledon. Most alkaloids have a complex ring structure and significant biological activity. Many well-known natural bioactive compounds are alkaloids, such as ephedrine and atropine, which have played a vital role in the treatment of diseases. BBR, DNLA, TET, and PRO have potential in DM and AD. Here, we review the role of alkaloids represented by BBR and DNLA in DM and AD and put forward ideas for the use of alkaloids in T3DM.

### Berberine

Berberine, one of the alkaloids extracted from a traditional Chinese herb, is mainly isolated from *Coptis chinensis, Berberis vulgaris, Hydrastis canadensis*, and *Phellodendron amurense* (Neag et al., 2018). BBR has shown some potential in the treatment of both DM and AD.

Berberine shows great potential in the treatment of DM. First, BBR dramatically reduces serum insulin levels and alleviates insulin resistance (Wang et al., 2018), working through promoting RXRA, reducing KCNQ1 and NR3C1 (Di et al., 2021), and attenuating palmitate-induced mitochondrial injury and apoptotic death. Moreover, BBR significantly prevents β-cell apoptosis and may improve islet β-cell function in T2DM (Li J. et al., 2019). Second, BBR upregulates glucokinase (GK) in liver fractions and liver glycogen content to an anti-diabetic effect by the dissociation of glucokinase GK from GK regulatory protein in db/db mice (Li M. et al., 2019). In addition, BBR is a substrate of P-gp. However, the oral bioavailability of BBR is less than 5%. Therefore, researchers use TET, another P-gp inhibitor, as an adjuvant component to potentiate the hypoglycemic efficacy of BBR (Shan et al., 2013). Finally, intestinal microbiota may serve as a potential target for berberine treatment of T2DM (Li et al., 2017). BBR could alleviate symptoms in T2DM rats by affecting gut microbiota composition and reducing the concentration of aromatic amino acids (Xu et al., 2020; Yao et al., 2020) such as decreasing the *Bacteroidetes, Bacteroidetes*/*Firmicutes* ratio, and *Muribaculaceae*, and increasing *Allobaculum* (Zhao et al., 2021). Clinical studies related to this also verified the effect. Furthermore, the hypoglycaemic effect of BBR is mediated by the inhibition of deoxycholic acid (DCA) biotransformation by *Ruminococcus bromii* (NCT0286126) (Zhang Y. et al., 2020). In summary, the anti-DM effect of BBR is related to affecting tissues such as the pancreas and liver and regulating intestinal flora.

At the same time, BBR also has potential in AD treatment. BBR may prevent the formation of NFTs and the disaggregation of Aβ in AD by limiting neuroinflammation and oxidative stress (Hussien et al., 2018; Akbar et al., 2021). *In vivo*, BBR rescues synapse damage and limits tau hyperphosphorylation in APP/PS1 mice possibly *via* inhibiting the NF-κB pathway and activating the liver kinase B1 (LKB1)/AMPK pathway, and these attenuate cognitive deficits (He et al., 2017; Cai et al., 2019). It also reduces APP and beta-site amyloid precursor protein cleaving enzyme 1 (BACE1) and facilitates Aβ clearance *via* autophagy *in vitro* (Huang et al., 2017). Furthermore, BBR may inhibit protein kinase (PKR)-like endoplasmic reticulum (ER) kinase (PERK)/eukaryotic initiation factor 2alpha (eIF2α) signaling-mediated BACE1 translation and attenuate ER stress (Liang et al., 2021). Apart from reducing the Aβ accumulation, BBR inhibits the apoptosis of neurons and promotes the formation of microvessels in the mouse brain by enhancing brain platelet endothelial cell adhesion molecule-1, vascular endothelial growth factor, etc. As the result, it promotes the formation of new vessels with a complete structure and perfect function, which in turn promoted the recovery of cerebral blood flow. Ultimately, it ameliorates cognitive deficits in 3 × Tg AD mice (Ye et al., 2021). *In vitro*, BBR protects neuronal cells against Aβ partly through lncRNA BACE1 antisense (BACE1-AS)/miR-132-3p axis, regulating the circular RNAs histone deacetylase 9 (circHDAC9)/miR-142-5p axis (Ge et al., 2020; Zhang N. et al., 2020). Meanwhile, it can inhibit Aβ-induced microglial activation *via* modulating the microglial M1/M2 activated state and the suppressor of cytokine signaling1 (SOCS1) mediates the process (Guo et al., 2021). In addition, it attenuates Aβ-induced neuronal damage by regulating miR-188/nitric oxide synthase 1 (NOS1) (Chen et al., 2020). Interestingly, BBR can also alleviate tau hyperphosphorylation and axonopathy associated with diabetic encephalopathy by regulating the PI3K/Akt/GSK-3β signaling pathway (Wang et al., 2018). Based on the role of BBR in DM and AD, it can be found that the basic anti-inflammation and anti-oxidation of BBR are the key to the protective effect, and the regulation of insulin-related signaling pathways and intestinal flora also plays an important role.

### *Dendrobium nobile* Lindl. alkaloids

As a pharmacologically active ingredient of Dendrobium *nobile Lindl*. DNLA was originally extracted from *D. nobile* Lindl, a traditional Chinese herbal medicine and medicinal material of Guizhou province, and has significant protective effects in T2DM and the nervous system, especially in AD.

Our previous studies show that DNLA reduces BG levels in animal models of T2DM such as db/db and KK-Ay mice, improves insulin resistance, and has a protective effect on pancreatic β cells of pancreatic islets in these animals (Zhang, 2016; Chen, 2018; Huang Q. et al., 2019). It increases the p-INSR level, IRS-1, and after that, activates Akt (Chen, 2018). Furthermore, telomere length is shortened in the pancreas of db/db mice, and DNLA can delay shortening telomere length and increase the telomerase activity. The action works may be related to upregulating *TERT, TERC* mRNA, protein expressions of TRF2, and POT1, and decreasing protein expression of TRF1 in the pancreas (Zhang, 2016). The key to the protective effect of DNLA in DM is to regulate the insulin signaling pathway and improve insulin resistance.

Our previous studies show DNLA can improve the neuronal disruption caused by LPS, oxygen-glucose deprivation, and reperfusion, and decrease neuronal apoptosis in the rat brain (Li L. S. et al., 2017; Zhang et al., 2017; Liu et al., 2021). Furthermore, we observed amelioration of the spatial learning performance in AD model rats induced by Aβ_25 − 35_, APP/PS1, and SAMP8 mice (Nie et al., 2016; Lv et al., 2020), and this effect may be related to a decrease in the generation of Aβ by regulating APP, α-secretases (ADAM10 and ADAM17), and BACE1 (Huang J. et al., 2019). It also alleviates Aβ_25 − 35_-induced axonal injury by improving autophagic flux in neurons, increasing Aβ clearance, activation of autophagy activity, and upregulation of Klotho (Li L. S. et al., 2017; Zhang et al., 2017; Lv et al., 2020), possibly *via* the suppression of ER stress-related PERK signaling pathway, sequentially inhibiting calpain 1, GSK-3β, and Cdk5 activities and eventually reducing the p-tau (Liu et al., 2020). Notably, DNLA improved learning and memory function in elderly normal mice. Based on the results of DNLA in DM and AD, it is shown that DNLA is a potential insulin sensitizer and has neuroprotective effects, which can significantly improve the learning and memory ability of AD model animals. Based on this, we speculate that the anti-AD effect of DNLA is mainly achieved by regulating insulin-related signaling pathways, thereby inhibiting the hyperphosphorylation of tau protein.

## Summary

**Figure 1:**
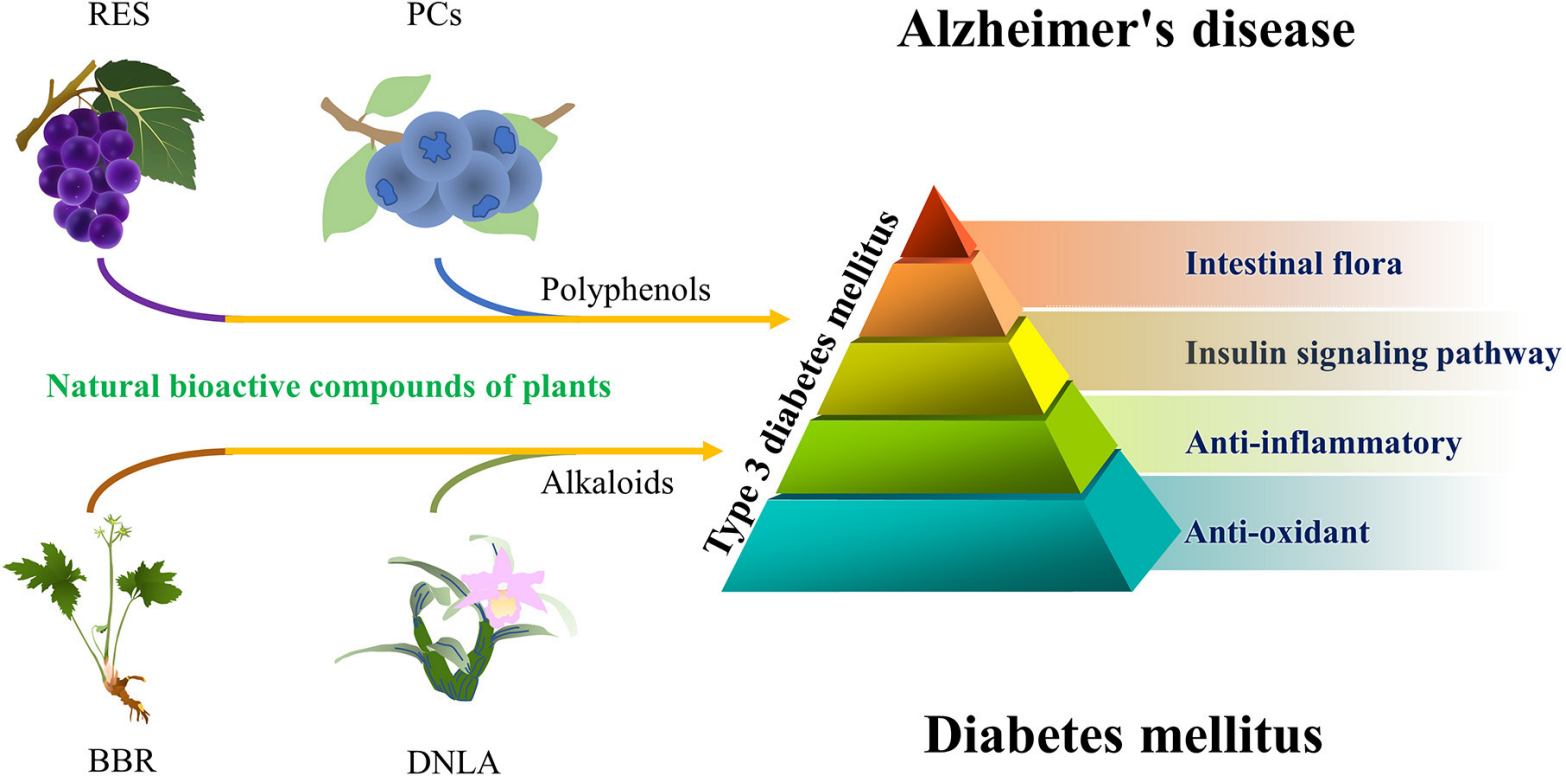
A simplified schematic diagram representing natural bioactive compounds in AD. From the perspective of T3DM. Grape—RES, blueberry—PCs, *Coptis chinensis*—BBR, and *Dendrobium nobile* Lindl.—DNLA.

In short, some of the natural bioactive compounds that have anti-DM effects have some potential in the treatment of AD. Their potential anti-AD ability is mainly based on anti-inflammation, anti-oxidation, regulation of insulin signaling pathway, and intestinal flora. These mechanisms are complex and involve pleiotropic synergistic interactions (Figure 1).

**Figure 2:**
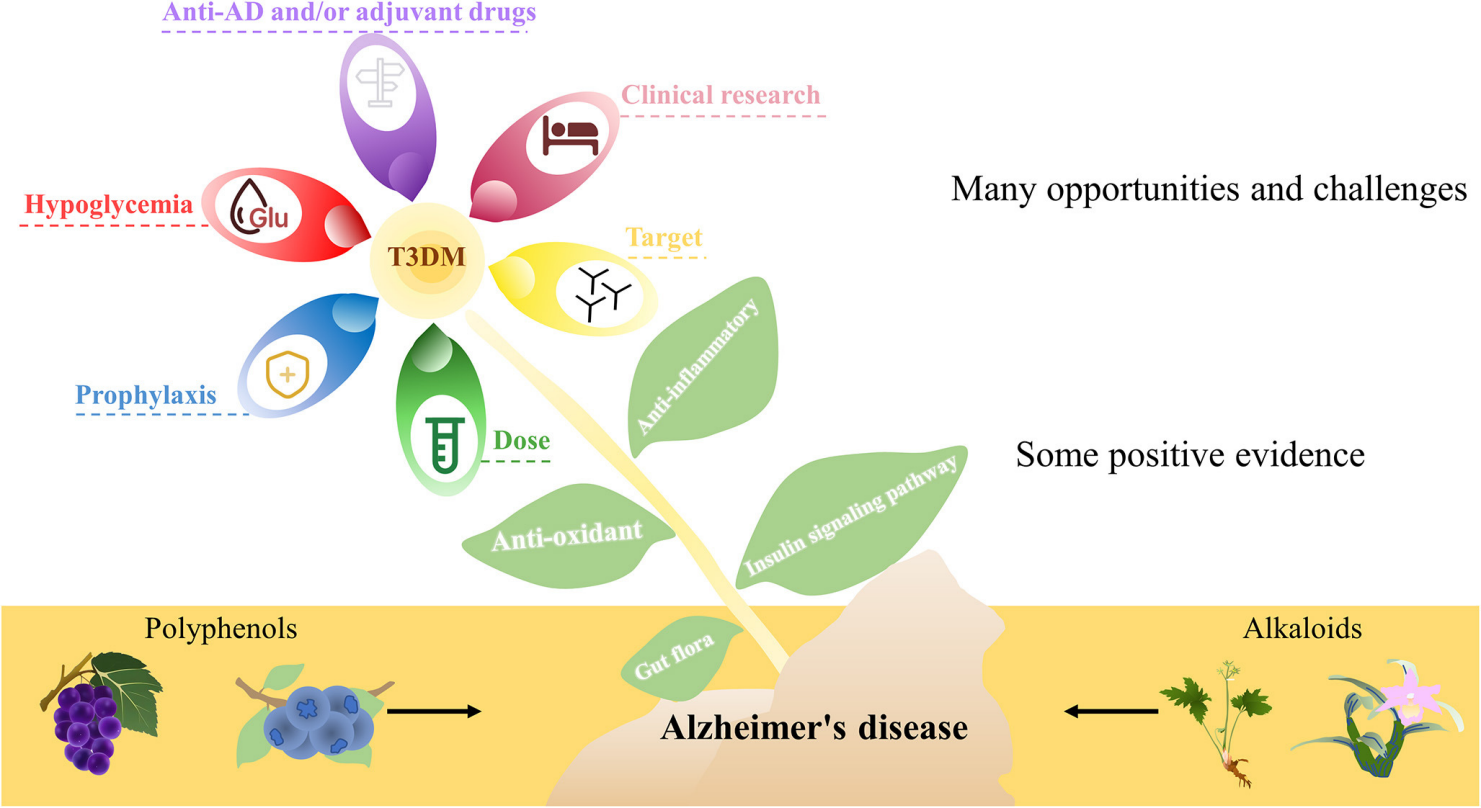
A simplified schematic diagram representing natural bioactive compounds in AD. Although there are some positive pieces of evidence, there are still many opportunities and challenges, and there are still many issues worthy of discussion.

Although there are some positive pieces of evidence, there are still many opportunities and challenges, and there are still many issues worthy of discussion. (1) Most of the studies in AD are preclinical studies and lack clinical research data. (2) Evidence from many studies is largely derived from non-target effects such as anti-inflammatory and antioxidant effects. (3) Unavoidable side effects, such as severe hypoglycemia, are even more harmful than AD. (4) The purpose of research and development of such drugs is direct anti-AD drugs and/or adjuvant drugs. (5) Many studies are carried out in the form of pretreatment, and it may be more reasonable to define it as prophylaxis. (6) Some studies only consider the experimental effect, ignoring the feasibility in humans after dose conversion (Figure 2).

Despite these problems, we must continue our research. We believe that the clinical research of such drugs should be aimed at some patients with AD, especially for some patients with AD with hyperglycemia as the early manifestation or with diabetes. In addition, in order to avoid excessive influence of non-target effects, it should be similar to the research and development process from guanidine hemisulfate to metformin. The structural modification of natural bioactive compounds could also avoid the occurrence of some side effects. Although bioactive compounds are a long way from being used in clinical drugs, they are still promising as functional food ingredients or adjuvant drugs for AD.

## Author contributions

JH, NH, QM, JS, and YQ contributed to the critical revision of the manuscript and read and approved the submitted version. All authors contributed to the article and approved the submitted version.

## References

[B1] Akbar M. Shabbir A. Rehman K. Akash M. S. H. Shah M. A. (2021). Neuroprotective potential of berberine in modulating Alzheimer's disease *via* multiple signaling pathways. J. Food Biochem. 45, e13936. 10.1111/jfbc.13936 34523148

[B2] Akimoto H. Negishi A. Oshima S. Wakiyama H. Okita M. Horii N.. (2020). Antidiabetic drugs for the risk of Alzheimer disease in patients with type 2 DM using FAERS. Am. J. Alzheimers Dis. Other Demen. 35, 1533317519899546. 10.1177/1533317519899546 32162525 PMC11005324

[B3] Alzheimer's Disease International (2019). World Alzheimer Report 2019: Attitudes to Dementia [Online]. Available online at: https://www.alz.co.uk/research/world-report-2019 (accessed September 20, 2019).

[B4] Asadi S. Moradi M. N. Khyripour N. Goodarzi M. T. Mahmoodi M. (2017). Resveratrol attenuates copper and zinc homeostasis and ameliorates oxidative stress in type 2 diabetic rats. Biol. Trace Elem. Res. 177, 132–138. 10.1007/s12011-016-0861-6 27744600

[B5] Bhatt J. K. Thomas S. Nanjan M. J. (2012). Resveratrol supplementation improves glycemic control in type 2 diabetes mellitus. Nutr. Res. 32, 537–541. 10.1016/j.nutres.2012.06.003 22901562

[B6] Biogen (2019). Biogen Plans Regulatory Filing for Aducanumab in Alzheimer's Disease Based on New Analysis of Larger Dataset from Phase 3 Studies [Online]. Available online at: http://investors.biogen.com/news-releases/news-release-details/biogen-plans-regulatory-filing-aducanumab-alzheimers-disease (Accessed October 22, 2019).

[B7] Bo S. Ponzo V. Evangelista A. Ciccone G. Goitre I. Saba F.. (2017). Effects of 6 months of resveratrol versus placebo on pentraxin 3 in patients with type 2 diabetes mellitus: a double-blind randomized controlled trial. Acta Diabetol. 54, 499–507. 10.1007/s00592-017-0977-y 28238190

[B8] Broderick T. L. Rasool S. Li R. Zhang Y. Anderson M. Al-Nakkash L.. (2020). Neuroprotective effects of chronic resveratrol treatment and exercise training in the 3xTg-AD mouse model of Alzheimer's disease. Int. J. Mol. Sci. 21, 7337. 10.3390/ijms21197337 33020412 PMC7582460

[B9] Cai Z. Y. Wang C. L. Lu T. T. Yang W. M. (2019). Berberine alleviates amyloid-beta pathogenesis *via* activating LKB1/AMPK signaling in the brain of APP/PS1 transgenic mice. Curr. Mol. Med. 19, 342–348. 10.2174/1566524019666190315164120 30873920

[B10] Capiralla H. Vingtdeux V. Zhao H. Sankowski R. Al-Abed Y. Davies P.. (2012). Resveratrol mitigates lipopolysaccharide- and Aβ-mediated microglial inflammation by inhibiting the TLR4/NF-κB/STAT signaling cascade. J. Neurochem. 120, 461–472. 10.1111/j.1471-4159.2011.07594.x 22118570 PMC3253186

[B11] Chen H. (2018). The effect of Dendrobium nobile Lindl. Alkaloids on Blood Glucose in KK-Ay Mice and the Mechanisms [Master]. Zunyi: Zunyi Medical University.

[B12] Chen J. Zhou Y. Mueller-Steiner S. Chen L. F. Kwon H. Yi S.. (2005). SIRT1 protects against microglia-dependent amyloid-beta toxicity through inhibiting NF-kappaB signaling. J. Biol. Chem. 280, 40364–40374. 10.1074/jbc.M509329200 16183991

[B13] Chen L. Sun P. Wang T. Chen K. Jia Q. Wang H.. (2012). Diverse mechanisms of antidiabetic effects of the different procyanidin oligomer types of two different cinnamon species on db/db mice. J. Agric. Food Chem. 60, 9144–9150. 10.1021/jf3024535 22920511

[B14] Chen M. Li L. Liu C. Song L. (2020). Berberine attenuates Aβ-induced neuronal damage through regulating miR-188/NOS1 in Alzheimer's disease. Mol. Cell. Biochem. 474, 285–294. 10.1007/s11010-020-03852-1 32779043

[B15] Chi T. C. Chen W. P. Chi T. L. Kuo T. F. Lee S. S. Cheng J. T.. (2007). Phosphatidylinositol-3-kinase is involved in the antihyperglycemic effect induced by resveratrol in streptozotocin-induced diabetic rats. Life Sci. 80, 1713–1720. 10.1016/j.lfs.2007.02.002 17346750

[B16] Di S. Han L. An X. Kong R. Gao Z. Yang Y.. (2021). *In silico* network pharmacology and *in vivo* analysis of berberine-related mechanisms against type 2 diabetes mellitus and its complications. J. Ethnopharmacol. 276, 114180. 10.1016/j.jep.2021.114180 33957209

[B17] Díaz G. Lengele L. Sourdet S. Soriano G. de Souto Barreto P. (2022). Nutrients and amyloid β status in the brain: a narrative review. Ageing Res. Rev. 81, 101728. 10.1016/j.arr.2022.101728 36049590

[B18] Durairajan S. S. K. Selvarasu K. Bera M. R. Rajaram K. Iyaswamy A. Li M.. (2022). Alzheimer's disease and other tauopathies: exploring efficacy of medicinal plant-derived compounds in alleviating tau-mediated neurodegeneration. Curr. Mol. Pharmacol. 15, 361–379. 10.2174/1874467214666210906125318 34488602

[B19] Fang Y. Su Z. Si W. Liu Y. Li J. Zeng P.. (2021). [Network pharmacology-based study of the therapeutic mechanism of resveratrol for Alzheimer's disease]. Nan Fang Yi Ke Da Xue Xue Bao 41, 10–19. 10.12122/j.issn.1673-4254.2021.01.02 33509748 PMC7867487

[B20] Feng L. Zhang L. (2019). Resveratrol suppresses Aβ-induced microglial activation through the TXNIP/TRX/NLRP3 signaling pathway. DNA Cell Biol. 38, 874–879. 10.1089/dna.2018.4308 31215797

[B21] Ge Y. Song X. Liu J. Liu C. Xu C. (2020). The combined therapy of berberine treatment with lncRNA BACE1-AS depletion attenuates Aβ(25-35) induced neuronal injury through regulating the expression of miR-132-3p in neuronal cells. Neurochem. Res. 45, 741–751. 10.1007/s11064-019-02947-6 31898085

[B22] Gong X. Xu L. Fang X. Zhao X. Du Y. Wu H.. (2020). Protective effects of grape seed procyanidin on isoflurane-induced cognitive impairment in mice. Pharm. Biol. 58, 200–207. 10.1080/13880209.2020.1730913 32114864 PMC7067175

[B23] Gonzalez-Abuin N. Pinent M. Casanova-Marti A. Arola L. Blay M. Ardevol A.. (2015). Procyanidins and their healthy protective effects against type 2 diabetes. Curr. Med. Chem. 22, 39–50. 10.2174/0929867321666140916115519 25245512

[B24] Guo Q. Wang C. Xue X. Hu B. Bao H. (2021). SOCS1 mediates berberine-induced amelioration of microglial activated states in N9 microglia exposed to β amyloid. Biomed. Res. Int. 2021, 9311855. 10.1155/2021/9311855 34778460 PMC8589517

[B25] Guo R. Liu B. Wang K. Zhou S. Li W. Xu Y.. (2014). Resveratrol ameliorates diabetic vascular inflammation and macrophage infiltration in db/db mice by inhibiting the NF-κB pathway. Diab. Vasc. Dis. Res. 11, 92–102. 10.1177/1479164113520332 24464099

[B26] Hamzé R. Delangre E. Tolu S. Moreau M. Janel N. Bailbé D.. (2022). Type 2 diabetes mellitus and Alzheimer's disease: shared molecular mechanisms and potential common therapeutic targets. Int. J. Mol. Sci. 23, 15287. 10.3390/ijms232315287 36499613 PMC9739879

[B27] He W. Wang C. Chen Y. He Y. Cai Z. (2017). Berberine attenuates cognitive impairment and ameliorates tau hyperphosphorylation by limiting the self-perpetuating pathogenic cycle between NF-κB signaling, oxidative stress and neuroinflammation. Pharmacol. Rep. 69, 1341–1348. 10.1016/j.pharep.2017.06.006 29132092

[B28] Huang D. D. Shi G. Jiang Y. Yao C. Zhu C. (2020). A review on the potential of Resveratrol in prevention and therapy of diabetes and diabetic complications. Biomed. Pharmacother. 125, 109767. 10.1016/j.biopha.2019.109767 32058210

[B29] Huang J. Huang N. Xu S. Luo Y. Li Y. Jin H.. (2020). Signaling mechanisms underlying inhibition of neuroinflammation by resveratrol in neurodegenerative diseases. J. Nutr. Biochem. 88, 108552. 10.1016/j.jnutbio.2020.108552 33220405

[B30] Huang J. Huang N. Zhang M. Nie J. Xu Y. Wu Q.. (2019). Dendrobium alkaloids decrease Aβ by regulating α- and β-secretases in hippocampal neurons of SD rats. PeerJ 7, e7627. 10.7717/peerj.7627 31534855 PMC6733236

[B31] Huang M. Jiang X. Liang Y. Liu Q. Chen S. Guo Y.. (2017). Berberine improves cognitive impairment by promoting autophagic clearance and inhibiting production of β-amyloid in APP/tau/PS1 mouse model of Alzheimer's disease. Exp. Gerontol. 91, 25–33. 10.1016/j.exger.2017.02.004 28223223

[B32] Huang Q. Liao X. Qin W. U. Wang P. Shi J. (2019). Effects of Dendrobium nobile Lindl. alkaloids on insulin resistance in diabetic rats with non-alcoholic fatty liver disease. Chin. J. Comp. Med. 29, 75–78.+98. 10.3969/j.issn.1671-7856.2019.08.012

[B33] Hussien H. M. Abd-Elmegied A. Ghareeb D. A. Hafez H. S. Ahmed H. E. A. El-Moneam N. A.. (2018). Neuroprotective effect of berberine against environmental heavy metals-induced neurotoxicity and Alzheimer's-like disease in rats. Food Chem. Toxicol. 111, 432–444. 10.1016/j.fct.2017.11.025 29170048

[B34] Imbimbo B. P. Ippati S. Watling M. Imbimbo C. (2023). Role of monomeric amyloid-β in cognitive performance in Alzheimer's disease: insights from clinical trials with secretase inhibitors and monoclonal antibodies. Pharmacol. Res. 187, 106631. 10.1016/j.phrs.2022.106631 36586644

[B35] Jia J. Wei C. Chen S. Li F. Tang Y. Qin W.. (2018). The cost of Alzheimer's disease in China and re-estimation of costs worldwide. Alzheimers Dement. 14, 483–491. 10.1016/j.jalz.2017.12.006 29433981

[B36] Labban S. Alghamdi B. S. Alshehri F. S. Kurdi M. (2021). Effects of melatonin and resveratrol on recognition memory and passive avoidance performance in a mouse model of Alzheimer's disease. Behav. Brain Res. 402, 113100. 10.1016/j.bbr.2020.113100 33417994

[B37] Lee S. J. Nam E. Lee H. J. Savelieff M. G. Lim M. H. (2017). Towards an understanding of amyloid-β oligomers: characterization, toxicity mechanisms, and inhibitors. Chem. Soc. Rev. 46, 310–323. 10.1039/C6CS00731G 27878186

[B38] Li C. He J. Z. Zhou X. D. Xu X. (2017). [Berberine regulates type 2 diabetes mellitus related with insulin resistance]. Zhongguo Zhong Yao Za Zhi 42, 2254–2260. 10.19540/j.cnki.cjcmm.20170307.014 28822177

[B39] Li J. Du H. Zhang M. Zhang Z. Teng F. Zhao Y.. (2019). Amorphous solid dispersion of Berberine mitigates apoptosis *via* iPLAβ/Cardiolipin/Opa1 pathway in db/db mice and in Palmitate-treated MIN6 β-cells. Int. J. Biol. Sci. 15, 1533–1545. 10.7150/ijbs.32020 31337982 PMC6643135

[B40] Li L. S. Lu Y. L. Nie J. Xu Y. Y. Zhang W. Yang W. J.. (2017). Dendrobium nobile Lindl alkaloid, a novel autophagy inducer, protects against axonal degeneration induced by Aβ(25-35) in hippocampus neurons *in vitro*. CNS Neurosci. Ther. 23, 329–340. 10.1111/cns.12678 28261990 PMC6492701

[B41] Li M. Dang Y. Li Q. Zhou W. Zuo J. Yao Z.. (2019). Berberine alleviates hyperglycemia by targeting hepatic glucokinase in diabetic db/db mice. Sci. Rep. 9, 8003. 10.1038/s41598-019-44576-7 31142783 PMC6541623

[B42] Li X. Sui Y. Wu Q. Xie B. Sun Z. (2017). Attenuated mTOR signaling and enhanced glucose homeostasis by dietary supplementation with lotus seedpod oligomeric procyanidins in streptozotocin (STZ)-induced diabetic mice. J. Agric. Food Chem. 65, 3801–3810. 10.1021/acs.jafc.7b00233 28314100

[B43] Liang Y. Ye C. Chen Y. Chen Y. Diao S. Huang M.. (2021). Berberine improves behavioral and cognitive deficits in a mouse model of Alzheimer's disease *via* regulation of β-amyloid production and endoplasmic reticulum stress. ACS Chem. Neurosci. 12, 1894–1904. 10.1021/acschemneuro.0c00808 33983710

[B44] Liu B. Huang B. Liu J. Shi J. S. (2020). Dendrobium nobile Lindl alkaloid and metformin ameliorate cognitive dysfunction in senescence-accelerated mice *via* suppression of endoplasmic reticulum stress. Brain Res. 1741, 146871. 10.1016/j.brainres.2020.146871 32380088

[B45] Liu Y. Pi T. Yang X. Shi J. (2021). Protective effects and mechanisms of *Dendrobium nobile* Lindl. alkaloids on PC12 cell damage induced by Aβ (25-35). *Behav. Neurol*. 2021, 9990375. 10.1155/2021/9990375 34447483 PMC8384511

[B46] Locatelli F. M. Kawano T. Iwata H. Aoyama B. Eguchi S. Nishigaki A.. (2018). Resveratrol-loaded nanoemulsion prevents cognitive decline after abdominal surgery in aged rats. J. Pharmacol. Sci. 137, 395–402. 10.1016/j.jphs.2018.08.006 30196020

[B47] Lu Z. Jia Q. Wang R. Wu X. Wu Y. Huang C.. (2011). Hypoglycemic activities of A- and B-type procyanidin oligomer-rich extracts from different Cinnamon barks. Phytomedicine 18, 298–302. 10.1016/j.phymed.2010.08.008 20851586

[B48] Lv L. L. Liu B. Liu J. Li L. S. Jin F. Xu Y. Y.. (2020). *Dendrobium nobile* Lindl. alkaloids ameliorate cognitive dysfunction in senescence accelerated SAMP8 mice by decreasing amyloid-β aggregation and enhancing autophagy activity. J. Alzheimers Dis. 76, 657–669. 10.3233/JAD-200308 32538851

[B49] Maria J. K. (2014). Proanthocyanidins, anthocyanins and cardiovascular diseases. Food Res. Int. 59, 41–52. 10.1016/j.foodres.2014.01.046

[B50] Michán S. Li Y. Chou M. M. Parrella E. Ge H. Long J. M.. (2010). SIRT1 is essential for normal cognitive function and synaptic plasticity. J. Neurosci. 30, 9695–9707. 10.1523/JNEUROSCI.0027-10.2010 20660252 PMC2921958

[B51] Moser C. Vickers S. P. Brammer R. Cheetham S. C. Drewe J. (2014). Antidiabetic effects of the *Cimicifuga racemosa* extract Ze 450 *in vitro* and *in vivo* in ob/ob mice. Phytomedicine 21, 1382–1389. 10.1016/j.phymed.2014.06.002 25022210

[B52] Moussa C. Hebron M. Huang X. Ahn J. Rissman R. A. Aisen P. S.. (2017). Resveratrol regulates neuro-inflammation and induces adaptive immunity in Alzheimer's disease. J. Neuroinflammation 14, 1. 10.1186/s12974-016-0779-0 28086917 PMC5234138

[B53] Neag M. A. Mocan A. Echeverría J. Pop R. M. Bocsan C. I. Crişan G.. (2018). Berberine: botanical occurrence, traditional uses, extraction methods, and relevance in cardiovascular, metabolic, hepatic, and renal disorders. Front. Pharmacol. 9, 557. 10.3389/fphar.2018.00557 30186157 PMC6111450

[B54] Nie J. Tian Y. Zhang Y. Lu Y.-L. Li L.-S. Shi J.-S.. (2016). Dendrobium alkaloids prevent Aβ25–35-induced neuronal and synaptic loss *via* promoting neurotrophic factors expression in mice. PeerJ 4, e2739. 10.7717/peerj.2739 27994964 PMC5157189

[B55] Ogura K. Ogura M. Shoji T. Sato Y. Tahara Y. Yamano G.. (2016). Oral administration of apple procyanidins ameliorates insulin resistance *via* suppression of pro-inflammatory cytokine expression in liver of diabetic ob/ob mice. J. Agric. Food Chem. 64, 8857–8865. 10.1021/acs.jafc.6b03424 27792335

[B56] Oliveira W. H. Braga C. F. Lós D. B. Araújo S. M. R. França M. R. Duarte-Silva E.. (2021). Metformin prevents p-tau and amyloid plaque deposition and memory impairment in diabetic mice. Exp. Brain Res. 239, 2821–2839. 10.1007/s00221-021-06176-8 34283253

[B57] Rawat P. Sehar U. Bisht J. Selman A. Culberson J. Reddy P. H.. (2022). Phosphorylated tau in Alzheimer's disease and other *Tauopathies* 23, 12841. 10.3390/ijms232112841 36361631 PMC9654278

[B58] Selvarasu K. Singh A. K. Iyaswamy A. Gopalkrishnashetty Sreenivasmurthy S. Krishnamoorthi S. Bera A. K.. (2022). Reduction of kinesin I heavy chain decreases tau hyperphosphorylation, aggregation, and memory impairment in Alzheimer's disease and tauopathy models. Front. Mol. Biosci. 9, 1050768. 10.3389/fmolb.2022.1050768 36387285 PMC9641281

[B59] Shan Y.-Q. Zhu Y.-P. Pang J. Wang Y.-X. Song D.-Q. Kong W.-J.. (2013). Tetrandrine potentiates the hypoglycemic efficacy of berberine by inhibiting P-glycoprotein function. Biol. Pharm. Bull. 36, 1562–1569. 10.1248/bpb.b13-00272 23924821

[B60] Simões-Pires C. Zwick V. Nurisso A. Schenker E. Carrupt P.-A. Cuendet M.. (2013). HDAC6 as a target for neurodegenerative diseases: what makes it different from the other HDACs? Mol. Neurodegener. 8, 7. 10.1186/1750-1326-8-7 23356410 PMC3615964

[B61] Sin T. K. Yung B. Y. Siu P. M. (2015). Modulation of SIRT1-Foxo1 signaling axis by resveratrol: implications in skeletal muscle aging and insulin resistance. Cell Physiol. Biochem. 35, 541–552. 10.1159/000369718 25612477

[B62] Sreenivasmurthy S. G. Iyaswamy A. Krishnamoorthi S. Reddi R. N. Kammala A. K. Vasudevan K.. (2022a). Bromo-protopine, a novel protopine derivative, alleviates tau pathology by activating chaperone-mediated autophagy for Alzheimer's disease therapy. Front. Mol. Biosci. 9, 1030534. 10.3389/fmolb.2022.1030534 36387280 PMC9643865

[B63] Sreenivasmurthy S. G. Iyaswamy A. Krishnamoorthi S. Senapati S. Malampati S. Zhu Z.. (2022b). Protopine promotes the proteasomal degradation of pathological tau in Alzheimer's disease models *via* HDAC6 inhibition. Phytomedicine 96, 153887. 10.1016/j.phymed.2021.153887 34936968

[B64] Steen E. Terry B. M. Rivera E. J. Cannon J. L. Neely T. R. Tavares R.. (2005). Impaired insulin and insulin-like growth factor expression and signaling mechanisms in Alzheimer's disease–is this type 3 diabetes? J. Alzheimers Dis. 7, 63–80. 10.3233/JAD-2005-7107 15750215

[B65] Steiner N. Balez R. Karunaweera N. Lind J. M. Münch G. Ooi L.. (2016). Neuroprotection of Neuro2a cells and the cytokine suppressive and anti-inflammatory mode of action of resveratrol in activated RAW264.7 macrophages and C8-B4 microglia. Neurochem. Int. 95, 46–54. 10.1016/j.neuint.2015.10.013 26522689

[B66] Szkudelska K. Deniziak M. Sassek M. Szkudelski I. Noskowiak W. Szkudelski T.. (2021). Resveratrol affects insulin signaling in type 2 diabetic goto-kakizaki rats. Int. J. Mol. Sci. 22, 2469. 10.3390/ijms22052469 33671110 PMC7957525

[B67] Szkudelski T. Szkudelska K. (2011). Anti-diabetic effects of resveratrol. Ann. N. Y. Acad. Sci. 1215, 34–39. 10.1111/j.1749-6632.2010.05844.x 21261639

[B68] The Lancet (2022). Lecanemab for Alzheimer's disease: tempering hype and hope. Lancet 400, 1899. 10.1016/S0140-6736(22)02480-1 36463893

[B69] Tolppanen A. M. Lavikainen P. Solomon A. Kivipelto M. Uusitupa M. Soininen H.. (2013). History of medically treated diabetes and risk of Alzheimer disease in a nationwide case-control study. Diabetes Care 36, 2015–2019. 10.2337/dc12-1287 23340883 PMC3687306

[B70] Tong B. C.-K. Huang A. S. Wu A. J. Iyaswamy A. Ho O. K.-Y. Kong A. H.-Y.. (2022). Tetrandrine ameliorates cognitive deficits and mitigates tau aggregation in cell and animal models of tauopathies. J. Biomed. Sci. 29, 85. 10.1186/s12929-022-00871-6 36273169 PMC9587578

[B71] Valencia-Hernandez L. J. Wong-Paz J. E. Ascacio-Valdés J. A. Chávez-González M. L. Contreras-Esquivel J. C. Aguilar C. N.. (2021). Procyanidins: from agro-industrial waste to food as bioactive molecules. Foods 10, 3152. 10.3390/foods10123152 34945704 PMC8701411

[B72] Vandal M. White P. J. Chevrier G. Tremblay C. St-Amour I. Planel E.. (2015). Age-dependent impairment of glucose tolerance in the 3xTg-AD mouse model of Alzheimer's disease. FASEB J. 29, 4273–4284. 10.1096/fj.14-268482 26108977

[B73] Wang S. He B. Hang W. Wu N. Xia L. Wang X.. (2018). Berberine alleviates tau hyperphosphorylation and axonopathy-associated with diabetic encephalopathy *via* restoring PI3K/Akt/GSK3β pathway. J. Alzheimers Dis. 65, 1385–1400. 10.3233/JAD-180497 30175975

[B74] Wang X. Sun G. Feng T. Zhang J. Huang X. Wang T.. (2019). Sodium oligomannate therapeutically remodels gut microbiota and suppresses gut bacterial amino acids-shaped neuroinflammation to inhibit Alzheimer's disease progression. Cell Res. 29, 787–803. 10.1038/s41422-019-0216-x 31488882 PMC6796854

[B75] Wu Q. Chen H. Lv Z. Li S. Hu B. Guan Y.. (2013). Oligomeric procyanidins of lotus seedpod inhibits the formation of advanced glycation end-products by scavenging reactive carbonyls. Food Chem. 138, 1493–1502. 10.1016/j.foodchem.2012.10.111 23411272

[B76] Xu J. Zheng T. Huang X. Wang Y. Yin G. Du W.. (2021). Procyanidine resists the fibril formation of human islet amyloid polypeptide. Int. J. Biol. Macromol. 183, 1067–1078. 10.1016/j.ijbiomac.2021.05.030 33965498

[B77] Xu X. Gao Z. Yang F. Yang Y. Chen L. Han L.. (2020). Antidiabetic effects of gegen qinlian decoction *via* the gut microbiota are attributable to its key ingredient berberine. Genomics Proteomics Bioinformatics 18, 721–736. 10.1016/j.gpb.2019.09.007 33359679 PMC8377040

[B78] Yao Y. Chen H. Yan L. Wang W. Wang D. (2020). Berberine alleviates type 2 diabetic symptoms by altering gut microbiota and reducing aromatic amino acids. Biomed. Pharmacother. 131, 110669. 10.1016/j.biopha.2020.110669 32937246

[B79] Ye C. Liang Y. Chen Y. Xiong Y. She Y. Zhong X.. (2021). Berberine improves cognitive impairment by simultaneously impacting cerebral blood flow and β-amyloid accumulation in an APP/tau/PS1 mouse model of Alzheimer's disease. Cells 10, 1161. 10.3390/cells10051161 34064687 PMC8150323

[B80] Yokozawa T. Lee Y. A. Cho E. J. Matsumoto K. Park C. H. Shibahara N.. (2011). Anti-aging effects of oligomeric proanthocyanidins isolated from persimmon fruits. Drug Discov. Ther. 5, 109–118. 10.5582/ddt.2011.v5.3.109 22466239

[B81] Zhang M. (2016). The *Effect* of Dendrobium nobile Lindl. Alkaloids on Blood Glucose in db/db Mice and the Mechanisms. Zunyi: Zunyi Medical University.

[B82] Zhang N. Gao Y. Yu S. Sun X. Shen K. (2020). Berberine attenuates Aβ42-induced neuronal damage through regulating circHDAC9/miR-142-5p axis in human neuronal cells. Life Sci. 252, 117637. 10.1016/j.lfs.2020.117637 32251633

[B83] Zhang W. Wu Q. Lu Y.-L. Gong Q.-H. Zhang F. Shi J.-S.. (2017). Protective effects of *Dendrobium nobile* Lindl. alkaloids on amyloid beta (25-35)-induced neuronal injury. Neural Regen. Res. 12, 1131–1136. 10.4103/1673-5374.211193 28852396 PMC5558493

[B84] Zhang Y. Gu Y. Ren H. Wang S. Zhong H. Zhao X.. (2020). Gut microbiome-related effects of berberine and probiotics on type 2 diabetes (the PREMOTE study). Nat. Commun. 11, 5015. 10.1038/s41467-020-18414-8 33024120 PMC7538905

[B85] Zhang Y. Huang N. Chen M. Jin H. Nie J. Shi J.. (2019). Procyanidin protects against 6-hydroxydopamine-induced dopaminergic neuron damage *via* the regulation of the PI3K/Akt signalling pathway. Biomed. Pharmacother. 114, 108789. 10.1016/j.biopha.2019.108789 30925459

[B86] Zhang Y. Huang N. Q. Yan F. Jin H. Zhou S. Y. Shi J. S.. (2018). Diabetes mellitus and Alzheimer's disease: GSK-3beta as a potential link. Behav. Brain Res. 339, 57–65. 10.1016/j.bbr.2017.11.015 29158110

[B87] Zhao J. D. Li Y. Sun M. Yu C. J. Li J. Y. Wang S. H.. (2021). Effect of berberine on hyperglycaemia and gut microbiota composition in type 2 diabetic Goto-Kakizaki rats. World J. Gastroenterol. 27, 708–724. 10.3748/wjg.v27.i8.708 33716449 PMC7934002

[B88] Zhao S. Zhang L. Yang C. Li Z. Rong S. (2019). Procyanidins and Alzheimer's disease. Mol. Neurobiol. 56, 5556–5567. 10.1007/s12035-019-1469-6 30649713

[B89] Zheng X. Zhu S. Chang S. Cao Y. Dong J. Li J.. (2013). Protective effects of chronic resveratrol treatment on vascular inflammatory injury in steptozotocin-induced type 2 diabetic rats: role of NF-kappa B signaling. Eur. J. Pharmacol. 720, 147–157. 10.1016/j.ejphar.2013.10.034 24436987

